# Cardiotoxicity of Antineoplastic Therapies and Applications of Induced Pluripotent Stem Cell-Derived Cardiomyocytes

**DOI:** 10.3390/cells10112823

**Published:** 2021-10-21

**Authors:** Mo-Fan Huang, Lon Kai Pang, Yi-Hung Chen, Ruiying Zhao, Dung-Fang Lee

**Affiliations:** 1 Department of Integrative Biology and Pharmacology, McGovern Medical School, The University of Texas Health Science Center at Houston, Houston, TX 77030, USA; mo-fan.huang@uth.tmc.edu (M.-F.H.); LonKai.Pang@bcm.edu (L.K.P.); 2 The University of Texas MD Anderson Cancer Center UTHealth Graduate School of Biomedical Sciences, Houston, TX 77030, USA; 3 Department of Neuroscience, Baylor College of Medicine, Houston, TX 77030, USA; 4 Department and Institute of Pharmacology, National Yang Ming Chiao Tung University, Taipei 112, Taiwan; conyjill014@gmail.com; 5 Center for Stem Cell and Regenerative Medicine, The Brown Foundation Institute of Molecular Medicine for the Prevention of Human Diseases, The University of Texas Health Science Center at Houston, Houston, TX 77030, USA; 6 Center for Precision Health, School of Biomedical Informatics, The University of Texas Health Science Center at Houston, Houston, TX 77030, USA

**Keywords:** stem cell, disease model, induced pluripotency, reprogramming, differentiation, chemotherapy, cancer, cardiotoxicity, personalized medicine, pharmacogenomics

## Abstract

The therapeutic landscape for the treatment of cancer has evolved significantly in recent decades, aided by the development of effective oncology drugs. However, many cancer drugs are often poorly tolerated by the body and in particular the cardiovascular system, causing adverse and sometimes fatal side effects that negate the chemotherapeutic benefits. The prevalence and severity of chemotherapy-induced cardiotoxicity warrants a deeper investigation of the mechanisms and implicating factors in this phenomenon, and a consolidation of scientific efforts to develop mitigating strategies. Aiding these efforts is the emergence of induced pluripotent stem cells (iPSCs) in recent years, which has allowed for the generation of iPSC-derived cardiomyocytes (iPSC-CMs): a human-based, patient-derived, and genetically variable platform that can be applied to the study of chemotherapy-induced cardiotoxicity and beyond. After surveying chemotherapy-induced cardiotoxicity and the associated chemotherapeutic agents, we discuss the use of iPSC-CMs in cardiotoxicity modeling, drug screening, and other potential applications. Improvements to the iPSC-CM platform, such as the development of more adult-like cardiomyocytes and ongoing advances in biotechnology, will only enhance the utility of iPSC-CMs in both basic science and clinical applications.

## 1. Induced Pluripotent Stem Cell-Derived Cardiomyocytes

Over the last 15 years, induced pluripotent stem cells (iPSCs) have emerged as a valuable tool to the research community. Human iPSCs are available in unlimited supply, on immediate demand, can be maintained for weeks to months in vitro, can be derived directly from patients to capture unique genetic signatures, and are amenable to genome modification and transfection operations. Since their relatively recent development by Kazutoshi Takahashi and Shinya Yamanaka [1], iPSCs continue to find new applications in disease modeling, mechanistic studies, drug development, biobanking, and therapeutic strategies across a diverse range of pathologies [2,3,4,5].

Notably, iPSCs have recently emerged as a platform for the study of chemotherapy-induced cardiotoxicity. Cancer is one of the leading causes of death globally; in 2020, there were an estimated 19.5 million new cases of cancer, and almost 10.0 million deaths due to cancer [6]. The continuing development of novel therapeutic agents has contributed to an improving trend of survival in cancer patients [7]. However, cardiotoxic side effects from chemotherapeutic agents remain common with deleterious consequences. Cardiotoxicity has been observed in multiple classes of chemotherapeutic agents, including anthracyclines, anti-microtubule agents, tyrosine kinase inhibitors (TKIs), and antibody-based drugs, such as trastuzumab [8]. Yet, the mechanisms of such cardiotoxicity are still poorly understood and unexpected in occurrence. The available clinical information is typically collected after the development of irreversible myocardial injury. For these reasons, chemotherapy-induced cardiotoxicity is still managed supportively following unpredictable onset, rather than preventively through mitigative measures [9].

Against this backdrop, iPSC-derived cardiomyocytes (iPSC-CMs) have begun demonstrating their value in the elucidation of chemotherapy-induced cardiotoxicity. iPSCs derived from patient cells (patient-derived iPSCs) capture the unique patient-specific genome while reprogramming mature cells (including cells exhibiting pathological genotypes) to a pluripotent state [10]. Differentiating these iPSCs to CMs through established protocols results in a stable cell culture, contrasting with adult CMs that cannot be reliably maintained in vitro [11,12]. iPSC-CMs are genetically identical to patients and somewhat similar to in vivo CMs. They express most of the cardiac-specific ion channels, boast a versatile contractile apparatus, possess calcium-handling properties, and beat spontaneously [8,9]. An alternative source for CMs is stem cell lines. Additionally, either of these stem cell sources can be genetically modified through gene editing tools such as CRISPR/Cas9 to introduce genes of interest [13].

These characteristics represent significant improvements to prior modeling platforms. Mouse models have been the mainstay in disease modeling and preclinical toxicity studies for decades, due to their high accessibility to entire organ systems and their amenability to genetic engineering. However, the substantial biological differences between animals and humans have led to low transferability of animal model-based findings to humans, as evidenced by the high attrition rate of drugs that make it from preclinical testing to final approval by the US Food and Drug Administration. Primary cells extracted directly from patients better recapitulate human physiology and disease traits, but these are often difficult to maintain in culture (sometimes due to pathogenic variants), and cellular sources are limited due to small patient sizes and difficulty of extraction (such as with cardiomyocytes). Human embryonic stem cells (hESCs) appeared to address many of these problems, being easily differentiable to any cell type and amenable to genetic engineering, but presented many ethical challenges due to how they are sourced. With this in mind, iPSCs such as iPSC-CMs represent the best modeling platform yet due to their human origin, easy accessibility, amenability to genetic engineering, and avoidance of ethical issues [11,12].

**Figure 1:**
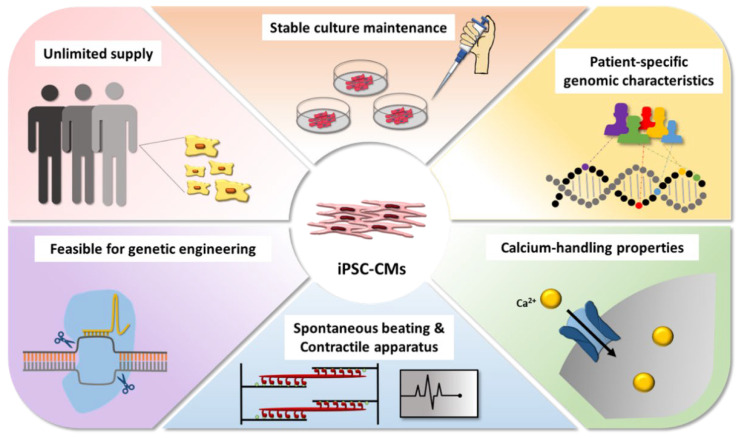
Features of induced pluripotent stem cell-derived cardiomyocytes (iPSC-CMs) that enhance their suitability for the modeling of anticancer therapy-associated cardiotoxicity. iPSC-CMs can be obtained in unlimited supply from patients, capturing the patient-specific genome while also allowing for genetic modifications. iPSC-CMs can be stable in culture for months, offering extended study times, while possessing unique cardiomyocyte phenotypes.

The applications of iPSC-CMs are diverse (Figure 1). Most directly, iPSC-CMs can be used for the screening of chemotherapeutic agents and the study of their cardiotoxic effects. Mechanistically, iPSC-CMs enable a closer study of the underlying genetic factors and biochemical pathways implicated in chemotherapy-induced cardiotoxicity, associating the unique characteristics of each drug to downstream implications. Such information can and has already been synthesized into in silico models for the prediction of cardiotoxicity, potentially informing future development of chemotherapeutic agents [14]. Clinically, iPSC-CMs can be used for appropriate dosage determination and the development of curative therapies to counteract drug-induced cardiotoxicity. iPSC-CMs are not without limitations, especially their resemblance to immature fetal CMs rather than mature adult CMs [9]. Still, iPSC-CMs have proven their value in the study of many cardiovascular disease, including LEOPARD syndrome [15], long QT syndrome [16], Brugada syndrome [17], LV non-compaction [18], dilated cardiomyopathies [19,20], and hypertrophic cardiomyopathies [21,22].

This review presents an overview of the use of iPSC-CMs in the modeling of chemotherapy-induced cardiotoxicity, beginning with an analysis of cardiotoxicity and chemotherapeutic agents implicated in cardiotoxicity, before exploring applications of iPSC-CMs to the field of chemotherapy and cardiotoxicity research.

## 2. Anticancer Therapy-Induced Cardiotoxicity

### 2.1. Epidemiology of Cardiotoxicity

The term “cardiotoxicity” as used in chemotherapeutic contexts often refer to myocardial dysfunction or heart failure, which are some of the most common and serious cardiotoxic effects [23,24,25]. Cardiotoxicity can be defined by the following criteria as determined by a heart echocardiograph: (1) an absolute decrease in the ejection fraction (EF) by 10% or more, and (2) an EF of less than 50%. A reduction by more than 15% in the left ventricular global longitudinal strain (GLS) has also been proposed as a predictor of left ventricular dysfunction, and may precede cardiotoxicity [26]. Clinical diagnostic methods include cardiac imaging modalities (such as echocardiographs, nuclear cardiac imaging, and cardiac magnetic resonance imaging) and biomarker screening (such as troponin and natriuretic peptides). Risk factors include current myocardial disease (such as ventricular dysfunction, heart disease, and cardiomyopathies), prior use of cardiotoxicity-associated chemotherapeutics (such as anthracyclines), and demographic and lifestyle factors (family history, tobacco and alcohol use). Primary clinical management strategies focus on reactive adjustments to treatments, such as reduction in dosage or substitution of drugs, and continual or preemptive surveillance [26].

Patient risk profiles, and drugs and classes of therapies used, are among many factors contributing to variations in time of onset, permanence, and complexity of cardiotoxic effects. For example, anthracycline-induced heart failure occurs at a rate of 0.2 to 8.7 percent depending on the cumulative dosage [27], while patients treated with trastuzumab experience heart failure at a rate of up to 3.8 percent [28].

In addition, anti-cancer therapies have been implicated in other cardiovascular complications including coronary artery disease or myocardial ischemia, valvular disease, arrhythmias, such as atrial fibrillation, arterial hypertension, thromboembolic disease, peripheral vascular disease and stroke, pulmonary hypertension, and pericardial complications. The risk factors and clinical diagnostic methods are largely similar to that of myocardial dysfunction, with echocardiography serving as a key imaging tool. Management strategies are reactive and symptom-focused, with minimal data supporting more substantive preventive or curative measures [26].

### 2.2. Molecular Mechanism of Anticancer Therapy-Induced Cardiotoxicity

**Figure 2:**
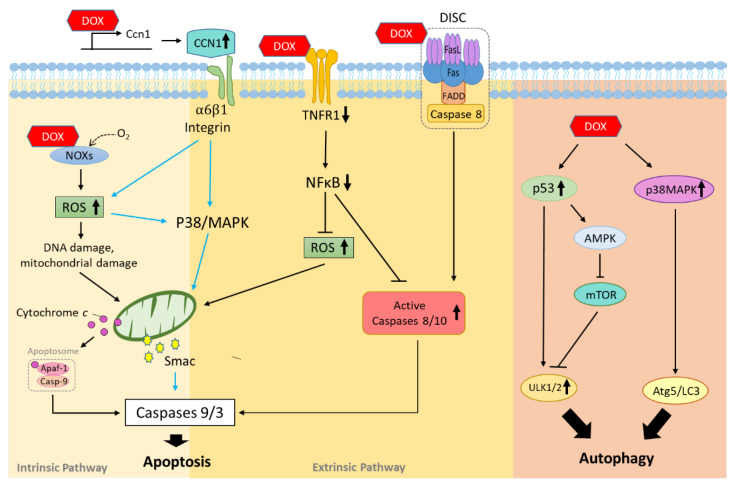
Mechanisms of doxorubicin implicated in chemotherapy-associated cardiotoxicity. Major doxorubicin-induced damages are caused through apoptosis and autophagy pathway. In apoptosis, doxorubicin affects apoptosis-related factors both in the intrinsic and extrinsic pathways. In autophagy, doxorubicin induces the activity of upstream modulators and triggers autophagy. Doxorubicin is represented as a red hexagon (DOX). Other factors are shown as colored ovals and rectangles.

Drug-induced cardiotoxicity can cause severe damage and cardiac disease such as arrhythmia, myocardial infarction, and myocardial hypertrophy, which could limit further use of the implicated drugs. Clinically available implicated drugs consist of anticancer drugs (doxorubicin, trastuzumab, cisplatin, etc.), antidiabetic drugs (pioglitazone, etc.), and an antiviral drug (zidovudine) [29,30]. Here, we introduce doxorubicin-induced cardiotoxicity and its underlying mechanisms based on prior research and history in clinical use. We also highlight key molecules and pathways associated with cardiomyocyte death signaling processes including apoptosis, autophagy, and necrosis (Figure 2).

#### 2.2.1. Intrinsic Apoptosis and Extrinsic Apoptosis

Apoptosis is the most widely studied form of cell death. Apoptosis exhibits cell death signs morphologically, such as loss or change in cell membrane permeability, increase in cytoplasmic density, shrinkage of cell size, or decrease in mitochondrial membrane potential. Eventually, dying cells become impaired apoptotic bodies and are degraded or absorbed in the cytoplasm. Based on known mechanisms, apoptosis can be intrinsic or extrinsic [31,32]. Intrinsic apoptosis can be caused by disruptions to the microenvironment, including excessive oxidative stress, DNA damage, endoplasmic reticulum (ER) stress, or mitochondria membrane disorders [33]. On the other hand, extrinsic apoptosis is initiated by plasma membrane receptors (e.g., FASL/FAS and TNFα/TNFR1) and death-inducing signaling complex (DISC), where death receptors initiate caspase reaction cascades [34].

Doxorubicin is known to increase production of reactive oxygen species (ROS) through many ways related to oxygen production, for example by affecting triphosphopyridine nucleotide (NADPH) oxidase [35]. Other studies found that chemical interactions between doxorubicin and NADPH increased superoxide formation, eventually causing DNA damage [36]. A previous study demonstrated that doxorubicin treatment induced cardiomyocyte apoptosis through mitochondrial apoptosis via caspase-3 induction and cytochrome C release pathways [37]. A pathway involving hypoxia-inducible factors is also responsible for the cardioprotective effect of dexrazoxane regulation against doxorubicin cardiotoxicity [38].

Doxorubicin is also known to facilitate binding events between death reporters and their corresponding ligands to facilitate DISC complex assembly, thereby activating caspase cascades. A matricellular protein CCN1 was found to mediate cardiotoxicity by engaging integrin a6β1 to promote the activation of mitogen-activated protein kinase (p38-MAPK), culminating in the release of second mitochondrial activator of caspase (SMAC) to induce cardiomyocyte apoptosis in mice [39]. TNFR1 is also involved in doxorubicin-induced cardiomyocyte death whereby changes in TNFα receptor expression modulated doxorubicin-induced H9c2 cardiomyocyte apoptosis via activation of caspase-8 and suppression of IκBα [40].

#### 2.2.2. Autophagy

Autophagy plays an essential role in maintaining intracellular metabolic homeostasis by degrading or consuming unwanted or damaged cellular components [41]. The mammalian target of rapamycin (mTOR) is an important signaling protein of autophagy as it complexes mammalian target of rapamycin complex 1 (mTORC1) with several other proteins stimulated by growth factor and receptor tyrosine kinase, blocking ULK-1-mediated Beclin 1 phosphorylation and stopping autophagy initiation [42]. Conversely, the adenosine 5-monophosphate activated protein kinase (AMPK) promotes autophagy by suppression of the mTOR-related complex and direct activation of ULK-1 phosphorylation [43].

In the context of doxorubicin, autophagy is mainly triggered by oxidative stress to protect cells against doxorubicin-induced cardiotoxicity. During doxorubicin treatment, levels of pro-autophagy factors (p53, p38-MAPK, and JNK-MAPK) increase while p85 expression decreases to attenuate the phosphoinosmde-3-kinase (PI3K) pathway [44,45]. Besides ROS-induced autophagy, doxorubicin also mediates autophagy-related factors and causes autophagy enhancement [46]. One recent study showed that downregulation of high mobility group box 1 (HMGB1) alleviates doxorubicin-induced cardiomyocyte damage by preventing autophagic cell death [46]. Nutritional deficiency or starvation prior to doxorubicin treatment was also found to decrease cardiotoxicity. For example, caloric restriction served a protective role by reducing ATP exhaustion and enhancing AMPK activity, thus attenuating the autophagy caused by doxorubicin [47].

#### 2.2.3. Necrosis

A third form of doxorubicin-induced cardiotoxicity cell death is necrosis, mostly believed to occur under higher doses or longer exposure periods to doxorubicin compared to apoptosis or autophagy. It has been reported that superoxide and peroxynitrite can increase necrosis in doxorubicin-induced cardiomyocyte death [48,49]. The typical dosage of doxorubicin is ≤20 mg/kg in vivo and 1 μM in vitro. Evidence of necrosis was observed in mice suffering from cardiac dysfunction following injections of 25 mg/kg doxorubicin [50], and in in vitro cardiac-derived H9c2 myocytes treated with 2 μM doxorubicin [51]. Moreover, initial apoptosis facilitated development into necrosis, where cells exhibited early DNA impairment and nuclear swelling as the exposure period increases [52]. These studies confirm that increased or prolonged doxorubicin dosage encourages necrosis.

### 2.3. Antineoplastic Agents Implicated in Cardiotoxicity

While many approved drugs are used for cancer treatment, some patients experienced adverse side effects following treatment. One of the most deleterious side effects is cardiovascular toxicity. Traditional and targeted chemotherapeutics are two major sources of cardiotoxicity. While the former can cause irreversible destruction to the myocardium, the latter can cause reversible damage to cellular functions and physiology [53,54,55]. Yet, differences in effects and dosage tolerances to the same drug between patients remain unexplained. In addition, the mechanisms of toxicity of these drugs can be diverse and multifactorial. Drugs that are known to be involved in toxicity can be classified in multiple ways; some of these categories include common chemotherapeutics such as anthracyclines, tyrosine kinase inhibitors (TKI), immune checkpoint inhibitors, and adoptive cellular therapy (ACT) (Table 1).

**Table 1:** Overview of chemotherapeutic agents and their effects as empirically documented both physiologically and in vitro in the iPSC-CM platform. The broad specificity of many chemotherapeutics result in unfavorable consequences on the cardiovascular system, ranging from benign asymptomatic structural heart damage, to both chronic presentations (such as hypertension, heart failure, and electrophysiological abnormalities), and acute presentations, such as acute ischemia.

Classes of Antineoplastic Agents	Cardiotoxic and Physiologic Effects	References
Anthracyclines	Long-term arrhythmia, cardiomyocyte dysfunction	[56,57,58,59,60,61,62,63]
(e.g., doxorubicin)		
Anti-metabolites	Coronary vascular spasms, structural heart damage (symptomatic and asymptomatic), systolic dysfunction, acute ischemia	[9,64]
(e.g., 5-fluorouracil)		
Alkylating agents	Structural heart damage (symptomatic and asymptomatic), systolic dysfunction, acute ischemia	[64,65]
(e.g., cyclophosphamide)		
Anti-microtubule	Systolic dysfunction, acute ischemia	[64]
(e.g., paclitaxel)		
Monoclonal antibodies	Ultrastructural changes, calcium dysregulation, mitochondrial dysfunction	[66,67,68]
(e.g., trastuzumab)		
Tyrosine kinase	Hypertension (systemic and pulmonary), myofibril dysfunction, fluid retention, QT prolongation	[61,62,69,70,71,72]
inhibitors		
(e.g., nilotinib)		
Proteasome inhibitors	Impaired left ventricular ejection fraction (LVEF), congestive heart failure (CHF)	[73,74]
(e.g., bortezomib)		
Immunomodulators	Sinus bradycardia, thromboembolic events	[75]
(e.g., thalidomide)		
Immune checkpoint	Myocarditis, cardiogenic shock, atrioventricular (AV) block, ventricular tachycardia	[76,77,78]
inhibitors		
(e.g., ipilimumab)		
Hormonal agents (e.g., letrozole)	Cardiac ischemia	[79]
Antiangiogenic agents (e.g., bevacizumab)	Hypertension, CHF, arterial thromboembolic events (ATEs)	[80]

#### 2.3.1. Chemotherapy Drug: Anthracycline

Although targeted tyrosine kinase- and monoclonal antibody-based therapies are recently developed, anthracyclines are still prescribed to 40–50% of breast cancer patients, along with other drugs, such as the alkylating agent cyclophosphamide, or the anti-microtubule agent taxanes, such as Paclitaxel (Taxol). Although doxorubicin has been clinically effective for a wide range of tumor cells, it imposes harsh effects on the human body through multiple cardiotoxicity mechanisms with both acute and chronic manifestations. Acute toxicity often happens immediately after anthracycline absorbance and manifests in myocardial ischemia, severe hypotension, and cardiac rhythm disturbance. New dosing guidelines have vastly reduced the incidence of acute cardiotoxicity. Still, chronic and sub-chronic cardiotoxicity remains a significant clinical problem. While sub-chronic toxicity may manifest in weeks or months, chronic toxicity may not surface until years or decades later. One study reported that children are more susceptible to the development of cardiomyopathy than adults given equivalent doses of daunomycin, implying the importance of the long-term monitoring of chronic cardiotoxicity [81].

The mechanisms behind anthracycline pharmacodynamics are complex. It has been suggested that anthracycline facilitates the excessive production of ROS and the activity of many ROS-dependent pathways such as double-stranded DNA damage response, protein synthesis attenuation, and mitochondrial dysfunction [82,83,84]. Further research revealed that deletion of topoisomerase II β (TOP2β) in mouse cardiomyocytes successfully prevented doxorubicin-induced cardiomyopathy, double-stranded breaks, and the formation of ROS [85]. On top of these, other unexpected cardiotoxic results are associated with doxorubicin exposure. Mouse models were used to study the molecular basis of doxorubicin-induced cardiotoxicity, revealing that high cumulative doses were associated with recalcitrant heart failure. This also resulted in a decline in cardiac systolic function, accompanied by marked atrophy of the heart, low levels of cardiomyocyte apoptosis, and decreased growth rate [86].

#### 2.3.2. Other Chemotherapy Drugs

Besides anthracycline compounds, other drugs including anti-metabolites and alkylating agents, platinum agents, and anti-microtubule agents have demonstrated their cardiotoxic potential. For example, 5-fluorouracil (5-FU) induces cardiotoxic effects, such as disturbances of rhythm and angina. 5-FU toxicity can lead to myocardial damage, vascular toxicity and vasospasm, the accumulation of toxic degradation products, and ROS-mediated metabolic damage. Less than 2% of patients treated with 5-FU experience more adverse effects such as congestive heart failure (CHF) [87,88]. The remaining classes of traditional chemotherapeutics have not showed cardiotoxic effects due to our limited understanding of their mechanisms of action. One anti-microtubule agent, taxanes, has been related to rhythm disturbances but the evidence for its cardiotoxicity is unsubstantial [8].

#### 2.3.3. Targeted Therapy Agents

The inhibition of growth-related kinases mediating cellular signal transduction, especially tyrosine kinases, is a new therapeutic strategy for diseases such as cancer. Studies have confirmed that the ATP-binding domain of tyrosine kinases is an attractive target for drug design and development. Both the EGFR and the HER2 receptor tyrosine kinase (RTK) inhibitors are effective drugs targeting solid tumors, and demonstrate higher targeting efficiency and reduced off-target effects compared to older treatments [89]. However, kinase inhibitors still cause adverse effects related to cardiovascular toxicity [90].

Among the HER2-positive metastatic breast cancer patients that reported trastuzumab cardiotoxicity, up to 27% of them experienced cardiac dysfunction and heart failure. Some presented with an increase in serum cardiac troponin I (cTnI) following mild reduction of left ventricular ejection fraction (LVEF) [91]. Combinatorial studies revealed that patients undergoing treatment with an anthracycline, cyclophosphamide, and trastuzumab concurrently had significantly increased risk of cardiac dysfunction compared to those only treated with an anthracycline and cyclophosphamide [92,93]. Recent investigations confirmed that trastuzumab disrupts HER2 signaling to mediate autophagy suppression, increases ROS production, and activates autophagy-inhibitory Erk/mTOR/Ulk 1 signaling [94]. Despite significant efforts to understand the molecular underpinnings of trastuzumab-induced cardiotoxicity, these mechanisms remain nebulous.

The tyrosine kinase inhibitor, imatinib, raised concerns of cardiotoxicity after 10 patients developed CHF following treatment in 2006 [95]. Human micrographs showed mitochondrial abnormalities and accumulation of membrane whorls in both vacuoles and the sarco-(endo-)plasmic reticulum after imatinib treatment. Because targeted kinases function through a similar mechanism, they may also induce similar toxicity issues. Currently, the FDA warning list of cardiotoxic drugs include six TKIs such as sunitinib, vemurafenib, and nilotinib. Sunitinib is associated with LVEF declines and QT prolongation [96]. These findings suggest the prevalence of off-target effects as primary causes of cardiotoxicity.

#### 2.3.4. Cancer Immunotherapy: Checkpoint Inhibitors and Adoptive Cellular Therapy

Immune checkpoint inhibitors and adoptive cellular therapies (ACT) are two categories of immunotherapies that play critical roles in the mitigation of cancer and autoimmune diseases. These cancer therapeutic strategies focus on activating and engaging the immune systems inherent in patients to destroy cancer cells [97,98]. Unfortunately, the advantages of immune checkpoint blocking antibodies can be accompanied by adverse cardiotoxic effects such as in cases of immune-related cardiotoxicity following treatment with ipilimumab, nivolumab, and/or pembrolizumab [99]. Documented cardiac dysfunction includes heart failure, cardiomyopathy, acute myocarditis, myocardial fibrosis, and pericarditis [100].

Furthermore, fatal adverse events, such as cardiac arrest and multiple organ failure, have been documented in patients treated with autologous T-cell receptor (TCR) transduced T cell infusion, an ACT [101]. Following MART-1 TCR transduced T cell treatment, high levels of NT-proBNP (a marker for heart failure) and IL-6 lead to diminished cardiac contractile function [102]. While the occurrence of immunotherapy-induced cardiotoxicity remains relatively low, these unfavorable side effects should be taken into consideration in therapeutic development and clinical applications.

## 3. Modeling Chemotherapy-Induced Cardiotoxicity with iPSC-CMs

iPSC-CMs are an excellent disease model to recapitulate the induction of cardiotoxicity by chemotherapeutic agents in cardiomyocytes. In particular, iPSC-CMs provide an unlimited cell source unlike many restrictions of animal models and primary cell lines. iPSC-CMs not only exhibit many cardiac-specific behaviors, including a cardiac contractile apparatus and calcium-handling features, but also demonstrate physiological and transcriptional responses after chemotherapy [103].

Several studies have established distinct technical approaches to model doxorubicin-induced effect on iPSC-CM’s transcriptomes, metabolisms, and functions (Table 2). Transcriptome analysis of doxorubicin-treated iPSC-CMs revealed the dynamic changes in global gene expression with respect to exposure time and dose [104]. Computational analysis of their transcriptomes further highlighted several signaling pathway genes associated with DNA damage and cell cycle regulation, including BLM, BRCA1, E2F, FANCG, PLK1, PRC1, and RBL1. E2F and RBL1 are known to control the estrogen-mediated S-phase entry pathway. FOXM1, p53, and E2F have been linked to left ventricular dysfunction, cardiomyocyte apoptosis, and heart failure, suggesting that the downregulation of PRC1 by transcription regulator FOXM1 may be a mediator of DNA damage repair response pathways induced by doxorubicin [105]. Furthermore, patient iPSC-CMs have been proved to recapitulate clinical observations in breast cancer patients and showed doxorubicin increases cellular ROS production, calcium handling, whole-cell oxidative stress, and eventually double–stranded DNA damage [56]. Transcriptome analyses of doxorubicin treatment on patient-specific gene expression reveal the significant downregulation of several important cardiac development-related transcription factors such as NKX2–5 (homeobox protein Nkx-2.5), MEF2A (myocyte-specific enhancer factor 2), and TBX5 (T-box transcription factor TBX5). Their study also revealed p53, RELA, NFKB1, and p300 play roles in doxorubicin pharmacodynamics and cardiotoxicity [56]. Kitani et al. discovered that the continued exposure of anti-cancer drugs, such as trastuzumab to iPSC-CMs impairs the contractile and calcium-handling abilities of cardiomyocytes without causing cell death or sarcomeric disorganization [66]. Transcriptome analysis suggested that mitochondrial functional defects and alterations to the cardiac energy metabolism pathway are primarily responsible for trastuzumab-induced cardiotoxic manifestations. Patient-derived iPSC-CMs that are more vulnerable to trastuzumab exhibited myocardial contractile dysfunction following treatment. Notably, activation of AMPK, a regulatory kinase in myocardial energy metabolism, could compensate for mitochondrial dysfunction, contractile dysfunction, and other adverse effects induced by trastuzumab. Maillet et al. engineered CRISPR/Cas9-mediated TOP2β deletions in hESC-CMs to investigate the role of TOP2β in doxorubicin-induced cardiotoxicity [13]. TOP2β-deleted NKX2-5eGFP/w hESC-derived CMs have similar cardiac gene expression with wild-type hESC-derived CMs but decrease drug susceptibilities in response to doxorubicin, indicating a critical role of TOP2β in doxorubicin-induced cardiotoxicity. In addition, Karhu et al. studied the chronic cardiotoxicity using iPSC-CMs under long-term low-dose administration of doxorubicin [106]. Their in vitro model showed that elevated caspase-3/7 activities are associated with decreased cell viability and increased apoptosis, and inhibition of GATA4 function provides cardioprotective effects in iPSC-CMs by attenuating the doxorubicin-induced elevation of pro-B-type natriuretic peptide expression. These results affirm that the iPSC-CM platform can be productively utilized for the evaluation of strategies to protect against or retroactively mitigate cardiomyocyte toxicity. These examples foreshadow the myriad potential applications of iPSC-CMs in cardiotoxicity studies, in vitro compound screening, and the wider field of synthetic biology.

**Table 2:** Use of iPSC-CMs in the study of anthracycline-induced cardiotoxicity. Numerous studies have successfully employed the iPSC-CM platform to elucidate the biochemical mechanisms of anthracycline pharmacodynamics, such as the implication of biomarkers, microRNAs, and genetic factors. The recent emergence of these studies suggest untapped potential in the field of iPSC-CM modeling. Studies organized chronologically.

Cardiotoxicity-Induced Drug	In-Vitro Observation Parameter: Functional Change Endpoint	In-Vitro Observation Parameter: Structural Change Endpoint	Application of iPSC-CMs	References
Daunorubicin	Beating frequency (xCELLigence)	Cell viability, ROS generation, Troponin secretion, lipid accumulation	Validation of appropriate parameters for testing DIC in iPSC-CMs	Doherty et al. [70]
Doxorubicin, Daunorubicin	Beating frequency (xCELLigence)	Troponin secretion and sarcomere structure.	Identification of biomarker from Doxorubicin-exposed iPSC-CMs global gene expression	Chaudhari et al. [57]
Doxorubicin	Multielectrode array (Maestro MEA system)	Cell viability, ROS generation, calcium handling, mitochondrial transmembrane potential, Apoptotic feature.	Investigation of the molecular mechanisms of DIC in a iPSC-CMs model system	Maillet et al. [13]
Doxorubicin	-	DNA damage level (γ-H2AX), calcium handling, ROS generation, mitochondrial function, Sarcomeric protein, apoptotic feature	Identification of the phenotype of DIC breast cancer patient-derived iPSC-CMs	Burridge et al. [56]
Doxorubicin	-	Lactate dehydrogenase (LDH) leakage	Identification microRNAs (miRNAs) expression from DIC iPSC-CMs	Chaudhari et al. [57]
Doxorubicin	Beating properties (Relaxation velocity, contraction velocity, contraction-relaxation duration, and beat rate) (Video microscopy)	Cardiac troponin, heart fatty acid-binding protein (FABP3), and N-terminal pro-brain natriuretic peptide (NT-proBNP)	Evaluation of the video microscopy approach in predicting chronic DIC in iPSC-CMs	Kopljar et al. [103]
Doxorubicin	Contractility (high-throughput contractility imaging)	Cytotoxicity	High-throughput contractility and cytotoxicity assay for cardiotoxicity induced drugs	Sharma et al. [107]
Doxorubicin	-	Cardiac troponin	Transcriptomic data from individual-derived iPSC-CMs	Knowles et al. [108]
Doxorubicin	-	-	Establishment of multi-omics data from Doxorubicin-exposed iPSC-CMs	Holmgren et al. [109]
Doxorubicin	Electrophysiological feature (cardiac optical mapping)	Cell viability, DNA damage level (γ-H2AX), ROS generation	In vitro correction of RARG mutation in patient-derived iPSC-CMs by CRISPR-Cas9	Christidi et al. [110]
Doxorubicin	-	Cell viability, pro-B-type natriuretic peptide (proBNP), Apoptotic feature	Chronic DIC iPSC-CMs in-vitro model for validating cardioprotective effect	Karhu et al. [106]

Abbreviation: DIC, doxorubicin-induced cardiotoxicity; ROS, reactive oxygen species.

## 4. Disease Modeling of iPSC-CM in Precision Medicine

**Figure 3:**
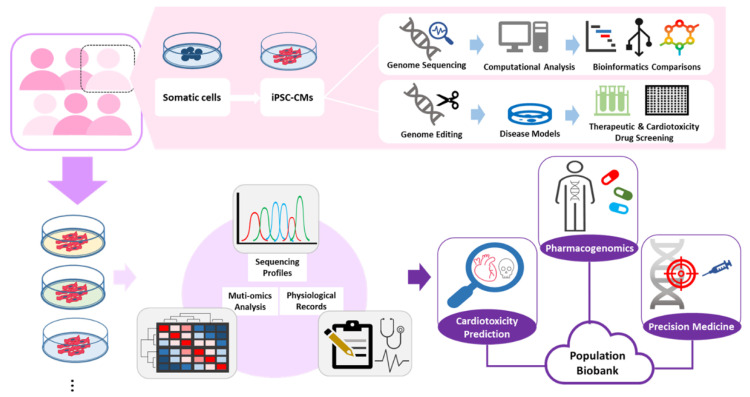
Generation and application of patient-derived iPSC-CMs. Somatic cells (fibroblasts) can be easily obtained from patients, reprogrammed into iPSCs, and differentiated into cardiomyocytes. In the individual iPSC-CMs model, iPSC-CMs provide a sophisticated resource to dissect chemo-induced cardiotoxicity as well as a useful platform for drug screening. With sufficient bioinformatics data and physiological records, abundant patient-derived iPSC-CMs models construct a substantial biobank that can be exploited in cardiotoxicity prediction, pharmacogenomics, and precision medicine.

The advancement of genetic analysis and modification techniques have paved the way for more precise study of the interactions between genetic variants, responses to chemotherapy, and cardiotoxicity consequences. Patient-derived iPSC-CMs are an excellent source of unique and personalized genomes that can now be meaningfully used to explore genetic associations in cardiotoxicity [111] (Figure 3).

### 4.1. iPSC-CM Disease Modeling in Studying Associations between Genetic Variations and Sensitivity to Cardiotoxicity

Several heterogenic background studies focusing on anthracycline-induced cardiotoxicity sensitivity and resistance have also been conducted through genome wide association studies (GWAS) and single nucleotide polymorphisms (SNPs) arrays in pediatric oncology cohorts. There were 18 significant genes carrying SNPs, which were known to be involved in DNA damage pathways, drug transport, oxidative stress defenses, or iron metabolism [112]. Missense mutations in the RARG gene were found to be potentially implicated in doxorubicin-induced cardiotoxicity. In one successful study, Christidi et al. employed CRISPR/Cas9 to generate isogenic iPSC-CMs with different RARG mutations, and discovered that doxorubicin-induced cardiotoxicity was reduced in iPSC-CMs with a PARG(S427L) mutation [110]. Another study found that RAC2 (which encodes Rho-GTPase to regulate the NADPH oxidase) and NADPH oxidase (NOX2) were both correlated with increased susceptibility to anthracycline-induced cardiotoxicity [113,114]. In the same way, the iPSC-CM model can be used to explore other SNPs and genetic predispositions associated with chemotherapy-induced cardiotoxicity.

In concert, these studies demonstrate that iPSC-CMs are effective not only in recapitulating the electrophysiological single-cell phenotype of cardiomyocytes, but also in demonstrating anthracycline-induced cardiotoxicity within a controlled and analyzable model system, allowing for the study of mechanisms underlying genetic disorders unique to the cardiovasculature.

### 4.2. Establishment of Personalized Cardiovascular Biobank for Toxicity Pre-Screening, Drug Testing, Therapeutics, and Diagnosis

The application of the iPSC-CM platform to therapeutics is promising. Using patient-derived iPSC-CMs in toxicity screens enable in-depth exploration of multiple candidate parameters associated with anticancer drug cardiotoxicity or drug efficacy. These studies can contribute meaningfully to the formulation of tailored, effective clinical treatments for each individual patient. The synthesis of data points into a clinical biobank can help with prediction and mitigation of harmful side effects and clinical advisory to reduce cardiovascular disease risk factors.

#### 4.2.1. Population Biobank for High-Throughput Toxicity Screening

A human cardiotoxicity biobank based on iPSC-CMs provides information on a wide range of clinical cardiotoxic effects, accounting for variations in severity and correlations between certain genetic variants and specific classes of targeted chemotherapeutic agents [115,116]. The biobank provides clinicians with a predictive model to make more informed decisions tailored to each patient, regarding the choice of chemotherapeutic agent, dosage, and other meaningful interventions. Promising toxicity trials to date have also inspired pharmaceutical companies to begin utilizing iPSC-CMs in tests for drug safety, arrhythmogenic potential, and other relevant parameters for preclinical drug testing.

Existing biobanks such as the GWAS Catalog provide accessible information on single nucleotide polymorphism (SNP)-trait associations, providing a springboard for researchers to investigate the impact of common variants on anthracycline-induced cardiotoxicity. A 2008 systematic review and meta-analysis gathered data from Medline, EMBASE, and the Cochrane Library on adults and children treated with an anthracycline for breast or ovarian cancer, sarcoma, non-Hodgkin’s or Hodgkin’s lymphoma, and myeloma. Their results included detailed information on defining and measuring cardiotoxicity outcomes, providing valuable perspectives that will inform research on anthracycline-induced cardiotoxicity research and the continued development of anthracycline chemotherapeutics [117]. This example foreshadows the insightful role that biobanks can play in toxicity screening for genetic factors.

#### 4.2.2. Pharmacogenomics

Pharmacogenetics is a field of study focused on the role of genetic variations in physiological responses to drugs. Age, environmental factors, and prior cardiovascular incidents contribute to drug response variability, but each patient’s genetic makeup may also predispose them towards certain cardiotoxic responses to chemotherapy [118]. With the growing awareness of precision medicine, pharmacogenetics has expanded to analysis of the entire human genome. On top of accounting for gene variants, pharmacogenomics also accounts for the unique biomarkers and levels present in each individual. For example, polymorphisms have been reported to have effects on susceptibility to chemotherapy-induced cardiotoxicity. Individual genetic variations may also affect the determination of treatment plans that balance drug efficacy and drug safety. Therefore, pharmacogenetic testing in chemotherapy-treated cancer patients is invaluable to understand the relationships between genetic variations and susceptibilities, and ultimately to facilitate the designing of personalized treatment strategies.

Advancements in next-generation sequencing (NGS) have significantly benefited the field of pharmacogenomics, facilitating an increase in the number of GWAS. GWAS aim to identify SNPs or the genetic loci associated with common diseases or traits. With the GWAS Catalog, oncologists can now better predict the drug response and any potential adverse drug reactions for each patient based on their genome [119]. A number of SNPs associated with doxorubicin-induced cardiotoxicity are documented [120]. However, validation studies for GWAS analysis are challenging due to their reliance on human cardiac biopsies, which are difficult to obtain. Conversely, the ease with which human patient iPSCs and disease-trait engineered iPSCs/hESCs can be cultured and extended make them the perfect disease platform to validate GWAS studies in a dish. In addition, iPSC-CMs can be applied in multi-omic analysis to investigate the links between genotypes and phenotypes in adverse drug reactions. Chaudhari et al. employed microarray statistical data analysis and functional annotation analysis to identify clusters of altered genes that potentially conferred doxorubicin sensitivity [57], suggesting the valuable contributions that iPSC-CM models can make to the field of pharmacogenomics. In sum, the iPSC-CM platform can synergize with biotechnological advancements to accelerate the growth of knowledge and consequently the development of breakthrough therapeutics.

## 5. Conclusions

The cardiotoxicity of chemotherapeutic compounds has been a major concern in the pharmaceutical and clinical fields. Efforts to minimize the harmful effects of chemotherapy on cancer patients would benefit greatly from a high-fidelity platform for disease modeling and drug screening. This review discussed the utility of iPSC-CMs for the study of drug-induced cardiotoxicity from multiple perspectives and the elucidation of the underlying mechanisms involved. The similarities between iPSC-CMs and physiological cardiomyocytes facilitate the screening of drug-induced alterations in cardiac cellular contractility, electrophysiology, and viability in ways previously inaccessible through animal models alone. In fact, high-throughput iPSC-CM models have facilitated the creation of a cardiac safety index (CSI) to grade drugs based on their potential cardiotoxicity and their quantitative toxicity measurements [107].

Patient-derived iPSC-CMs show great potential in the field of personalized medicine. Patient-specific iPSC-CMs can be used to identify genetic mutations that predispose toward or against cardiotoxicity, discover drugs for the treatment of both cancer and other cardiovascular diseases, and accumulate biobanks of data for the development of predictive models of efficacy and toxicity. Yet, challenges exist in the standardization and reproducibility of iPSC-CM generation, and the immature phenotype of iPSC-CMs. Future efforts should focus on the development of established and reproducible experimental protocols, and the improvement of iPSC-CMs to more accurately model mature adult cardiomyocytes. Still, iPSC-CMs will continue to be an excellent platform for the study of chemotherapy-induced cardiotoxicity and a plethora of other applications in disease modeling, toxicity screening, pharmacogenetics, and synthetic biology in general.

## Data Availability

Not applicable.
